# Delay time of waves performing Lévy walks in 1D random media

**DOI:** 10.1038/s41598-020-77861-x

**Published:** 2020-11-30

**Authors:** L. A. Razo-López, A. A. Fernández-Marín, J. A. Méndez-Bermúdez, J. Sánchez-Dehesa, V. A. Gopar

**Affiliations:** 1 Instituto de Física, Benemérita Universidad Autónoma de Puebla, Apartado Postal J-48, Puebla, 72570 Mexico; 2 Departamento de Ingeniería Electrónica, Universitat Politècnica de València, Camino de vera s. n. (Edificio 7F), 46022 Valencia, Spain; 3 Departamento de Física Teórica, Facultad de Ciencias, BIFI, Universidad de Zaragoza, Pedro Cerbuna 12, 50009 Zaragoza, Spain; 4 Present Address: Institut de Physique de Nice, CNRS, Universitè Côte d’Azur, Parc Valrose, 06100 Nice, France

**Keywords:** Materials science, Optics and photonics, Physics

## Abstract

The time that waves spend inside 1D random media with the possibility of performing Lévy walks is experimentally and theoretically studied. The dynamics of quantum and classical wave diffusion has been investigated in canonical disordered systems via the delay time. We show that a wide class of disorder—Lévy disorder—leads to strong random fluctuations of the delay time; nevertheless, some statistical properties such as the tail of the distribution and the average of the delay time are insensitive to Lévy walks. Our results reveal a universal character of wave propagation that goes beyond standard Brownian wave-diffusion.

## Introduction

A wave packet launched into a scattering region can penetrate that region and it may be reflected eventually. Thus, one might wonder how much time the wave packet has spent inside the media. This fundamental question was addressed by Wigner and Smith^1,2^. It was shown that the delay time $\tau _R$ of a wave packet is related to the derivative of the reflection phase $\theta _R$ with respect to the frequency $\omega$: $\tau _R = d\theta _R/d\omega$.

The delay time has received attention in many disciplines since it reveals information on the scattering medium and, therefore, it has also been of interest from an application point of view; e.g., the delay time is a fundamental quantity in imaging of tissues in optical coherence tomography^3,4^.

A major issue in wave transport is the presence of disorder. Moreover, if waves propagate coherently through 1D random media, complex interference effects emerge, such as the widely studied phenomenon of Anderson localization^5,6^: an exponential decay in space of classical and quantum waves, for instance, electromagnetic waves and electrons, respectively.

Since disorder is ubiquitous in real systems, there has been a great interest in studying the effects of Anderson localization on dynamical quantities such as the delay time. Microwave experiments have been performed to analyze statistical properties of wave dynamics^7–9^, while several theoretical approaches have been developed to describe the delay-time statistics (see Ref.^10^ for a review).

Remarkably, it has been demonstrated that some statistical properties of the delay time are invariant in the sense that they are independent of the details of the medium. For instance, the inverse square power decay of the distribution of $\tau _R$ has been predicted in semi-infinite 1D systems^11^ and also studied in higher dimensions^12–14^. Another example is the average delay time, which is proportional to the mean length of trajectories^15^. This quantity was predicted to be invariant with respect to details of the scattering region, as recently observed experimentally^15–17^.

**Figure 1:**
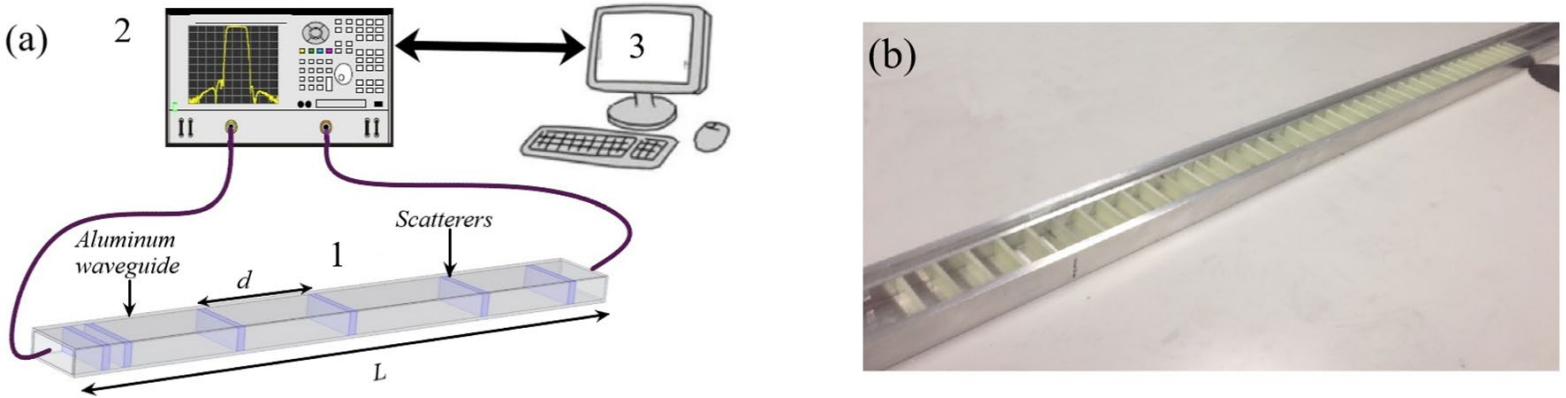
**(a)** Schematic view of the experimental setup. The aluminum waveguide (1), containing randomly distributed dielectric slabs, is connected to the ports of a vector network analyzer (2) and the data is stored in a computer (3).**(b)** Actual 2 m long aluminum waveguide (22.8 mm width and 10.6 mm height). The top is open to allow inner vision.

Previous experimental and theoretical works on the delay time in 1D consider only models of disorder that lead to Anderson localization, however, there is a wide class of disorder—Lévy disorder—that leads to delocalization or anomalous localization, in relation to the Anderson localization^18–21^. Anomalous localization finds its origin in the nonzero probability that waves travel a long distance without being scattered; these events are scarce but have a large impact. Such kind of events are sometimes called Lévy flights, however, the term Lévy flights is mostly used to study processes where the jumps or flights are instantaneous, while in Lévy walks, the velocity of the flights is constant, as in our study.

Here, we experimentally and theoretically study the delay time of reflected microwaves suffering coherent multiple scattering in a medium characterized by random spacings of scatterers following a one-sided Lévy $\alpha$-stable distribution. Experiments in waveguides with standard disorder (i.e., with random scatterer separations following a non-heavy tailed distribution) are also performed to compare results with those of Lévy disorder. Additionally, numerical simulations are carried out to overcome some practical limitations of experiments and to obtain further support for our model. We calculate the distribution of the delay time and, furthermore, our results allow us to conclude that universal features of the delay time in canonical disordered media go beyond standard Brownian models of wave diffusion, despite the fact that the presence of Lévy walks leads to stronger random fluctuations of the delay time.

Lévy statistics have been found in a broad range of contexts that go from foraging patterns of marine predators^22^ to fluctuations of stock market indices^23^ or resonant emission of light^24^. Lévy models are applied to describe anomalous diffusion of particles and waves that cannot be described by standard Brownian models^25–32^.

Essentially, Lévy random processes are characterized by probability distributions whose tails decay like a power-law, i.e., if *x* is a random variable with probability density $\rho (x)$, then $\rho (x) \sim 1/x^{1+\alpha }$ for $x \gg 1$^33^. Fluctuations of random variables that follow Lévy statistics are so large that the first and second moments diverge for $0< \alpha <1$.

## Results

### Experiments

Microwaves are launched into an aluminum waveguide containing 2.5 mm thick dielectric slabs whose separations follow a Lévy $\alpha$-stable distribution (see Fig. 1). We work in a frequency range where a single transport channel is supported. Two different $\alpha$-stable distributions characterized by their power-law decay have been chosen: $\alpha =1/2$ and 3/4. Additionally, a conventional disordered microwave waveguide with random spacing between slabs following a Gaussian distribution has been built. Using a network vector analyzer, we measure the $2 \times 2$ scattering matrix *S*:

1 $$\begin{aligned} S= & {} \left( \begin{array}{cc} \sqrt{R} e^{i\theta _R} &{} \sqrt{T} e^{i\theta _T} \\ \sqrt{T} e^{i\theta _T} &{} \sqrt{R}e^{i\theta _{R'}} \\ \end{array} \right) , \end{aligned}$$

where *R* and *T* are the reflection and transmission coefficients, respectively. Measurements of *S* are thus collected over different disorder realizations.

**Figure 2:**
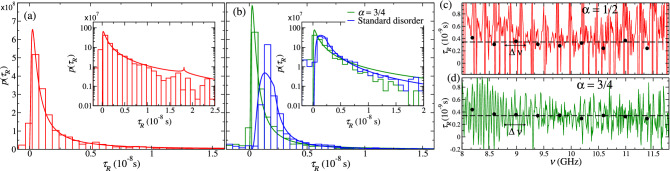
Experimental delay-time distributions (histograms) for Lévy waveguides characterized by **(a)** $\alpha =1/2$ and $\left\langle -\ln T \right\rangle =4.7$ at 9.9 GHz (red histogram) and **(b)** $\alpha =3/4$ and $\left\langle -\ln T \right\rangle =12$ at 11.2 GHz (green histogram). The blue histogram in **(b)** corresponds to random waveguides with ordinary (Gaussian) disorder with $\left\langle -\ln T \right\rangle =12$ at 11.2 GHz. The histograms were constructed with **(a)** 4590 and **(b)** 1890 data. Insets show $p(\tau _R)$ on a logarithmic scale. Red, green (blue) solid curves show the theoretical predictions from Eq. (4) (Eq. (3)). Experimental delay times for a typical realization of the disorder of waveguides with **(c)** $\alpha =1/2$ and **(d)** 3/4. Black dots represent the average of $\tau _R$ over frequency windows $\Delta \nu =0.4$ GHz. The horizontal black dashed lines are the averages of $\tau _R$ over the complete frequency window (8–12 GHz).

From the collected *S*-matrices, we obtain $\tau _R$ and the probability distribution function $p(\tau _R)$. Figure 2a,b show the distribution $p_\alpha (\tau _R)$ with $\alpha =1/2$ (red) and 3/4 (green), respectively. The insets show $p_\alpha (\tau _R)$ on a logarithmic scale for a better visualization of the tail. In Fig. 2b, the delay-time distribution (blue histogram) for conventional disorder is also shown. Both distributions in Fig. 2b have the same average value $\langle \ln T \rangle$. We can observe that the profile of both distributions (green and blue histograms) is different.

The distributions $p_\alpha (\tau _R)$ from our model, which we introduce below, are also shown (solid lines) in Fig. 2a,b. It is observed in Fig. 2a that for $\alpha =1/2$, $p_\alpha (\tau _R)$ shows a small peak in the tail. The distribution for $\alpha =3/4$ in Fig. 2b also exhibits a peak, but it is smoother and occurs at a larger value of $\tau _R$, outside of the time range shown in Fig. 2b. We attribute these peaks to scattering processes that reach the right boundary of the waveguide; in Lévy disordered samples those processes are favored since waves can travel long distances without being scattered. In contrast, for ordinary disordered systems, $p(\tau _R)$ decays monotonically and for $\tau _R \gg 1$, $p(\tau _R) \sim 1/\tau _R^{2}$^11^.

The trend of the experimental distributions (histograms) is well described by the model (solid lines), despite the fact that the statistics of $\tau _R$ is extracted from a limited amount of experimental data and the presence of a small tail for negative values of $\tau _R$ observed in Fig. 2a,b. Negative delay times are not considered in our model and are thus a source for discrepancies between experimental and theoretical results. It has been proposed that those negative values are due to a strong distortion of the wave packet produced due to interference between incident and promptly reflected waves^34–37^.

We now address an invariance property of the mean path of trajectories with respect to the details of the disordered medium. This invariance property is equivalent to the independence of the average delay time with energy since both quantities are proportional^15^. To illustrate this invariance, in Fig. 2c,d, $\tau _R$ is plotted as a function of the linear frequency $\nu$ for typical samples with $\alpha =1/2$ and 3/4, respectively. We see strong fluctuations of $\tau _R$, however, the average delay-time over frequency windows $\Delta \nu (=0.4$GHz) is essentially independent of the frequency, as it is observed in both figures (dots). Moreover, the average over the whole frequency window (horizontal dashed line) has the same value ($3.4\times 10^{-9}$s) for both cases: $\alpha =1/2$ and 3/4, and thus, it is independent of particularities of the medium. We will address this point later in more detail.

### Model

For conventional disorder and within a random matrix approach to localization^38,39^, the mean free path $\ell$ determines the statistical properties of the transport and it is related to the transmission by $s \equiv \langle -\ln T \rangle =L/\ell$, which is proportional to the number of scatterers *n* in the system, i.e., $\langle -\ln T \rangle = b n$ with *b* constant^40^. If the random spacing between scatterers follows a Lévy $\alpha$-stable distribution, the number of scatterers in a system of length *L* is subject to strong random fluctuations. Such fluctuations are described by the probability density $\Pi _L(n;\alpha )$ given by^18^: $\Pi _L(n;\alpha )=2L q_{\alpha ,1}\left( L/(2n)^{1/\alpha }\right) /\alpha (2n)^{(1+\alpha )/\alpha }$, where $q_{\alpha ,c}(x)$ is the probability density function of the Lévy $\alpha$-stable distribution with exponent $\alpha$ and scale parameter *c*. For $x \gg 1$, $q_{\alpha ,c}(x) \sim c/x^{1+\alpha }$. Therefore, with the knowledge of $\Pi _L(n;\alpha )$ and the delay-time probability density for canonical disordered systems, $p_s (\tau _R)$, we write the probability density $p_\alpha (\tau _R)$ for Lévy disordered systems as

2 $$\begin{aligned} p_\alpha (\tau _R)=\int _0^{\infty }p_s(\tau _R) \Pi _L(n;\alpha ) dn. \end{aligned}$$

The probability density $p_s(\tau _R)$ in its full generality remains, however, an open problem. For semi-infinite disordered systems, assuming no transmission, the limit ($L \rightarrow \infty$) delay-time distribution $p_{\infty }(\tau _R)$ is given by^11,41–45^: $p_{\infty }(\tau _R) = \tau _\ell \tau _R^{-2} \exp {(-\tau _\ell /\tau _R)}$, where $\tau _\ell$ is the scattering time of the disorder. Real systems, however, are finite and finite-size effects may be of relevance. In particular, our experiments are performed in 2 m long waveguides and microwaves can be transmitted.

Our model for $p_s(\tau _R)$ that will be verified experimentally and numerically involves two main assumptions. Firstly, though the waves in our waveguides can be transmitted, we use a relationship between $\tau _R$ in the absence of absorption and *R* in the presence of weak absorption that assumes negligible transmission: $R=1-\tau _R/\tau _0$, where $\tau _0$ is the absorption time which is assumed very large^45–47^. A key point is that this relationship establishes that fluctuations of *R* determine the statistics of $\tau _R$. Notice that for an ensemble of different samples, $\tau _0$ fluctuates since it depends on the disorder configuration. Indeed, later $\tau _0$ will be identified with $\langle \tau _R \rangle$, which contains information about the system length. Secondly, we use the Laguerre ensemble $p(\mu )$ which describes the statistics of *R* assuming large samples^48^. We have verified that the Laguerre ensemble describes well our experimental results for the reflection of canonical disordered waveguides even though they are 2m long. See supplemental material (SM). That is, $p(\mu ) \propto \exp {\left( - \gamma \mu \right) }$ where $\mu ^{-1} \equiv R-1$ and $\gamma$ is a constant. For systems with absorption ($R < 1$) $\gamma < 0$ and $\mu < -1$, while for systems with amplification ($R>1$), $\gamma > 0$ with $\mu >0$. For the latter case $R=1+\tau _R/\tau _0$. Let us stress that for systems of finite length, $\tau _R$ can exceed $\tau _0$ since, for instance, $\tau _R$ is infinite for transmitted waves. We thus need to consider both cases $\tau _R < \tau _0$ and $\tau _R > \tau _0$. Therefore, after the change of variable $\mu \rightarrow \tau _R$, the normalized distribution $p_s(\tau _R)$ can be expressed as

3 $$\begin{aligned} p_s(\tau _R) = \frac{a}{s \left[ 2-\exp {(-a/s)}\right] }\frac{1}{\tau _R^2} \exp \left( {-a \left| 1/\tau _R -1\right| /s} \right) , \end{aligned}$$

where $a\equiv 2L/(v_g \tau _0)$ and $v_g$ is the group velocity. In writing Eq. (3), we conveniently measure the delay time in units of $\tau _0$, i.e., we replaced $\tau _R/\tau _0 \rightarrow \tau _R$. The above expression for $p_s(\tau _R)$ is obtained after making $\mu \rightarrow |\mu +1|$ and the change of variable $\mu \rightarrow \tau _R$ in the Laguerre ensemble $p(\mu )$ with $-\mu ^{-1}=1-R=\tau _R/\tau _0$. Notice that with the absolute value in Eq. (3), both cases $\tau _R/\tau _0<1$ and $\tau _R/\tau _0>1$ are considered. Our assumptions may overestimate $p_s(\tau _R)$, mainly for $\tau _R > \tau _0$, since some of the scattering processes that reach the right end of the sample may leave the waveguide, having actually an infinite reflection delay time. Thus, as the systems become shorter, discrepancies between our model and experiments or simulations are expected. On the other hand, Eq. (3) reduces to $p_{\infty }(\tau _R)$ for $\tau _0/\tau _R \gg 1$. Additionally, since Eq. (3) is based on the Laguerre ensemble, in the SM we have experimentally and numerically verified the distribution of the reflection coefficient predicted by the Laguerre ensemble.

**Figure 3:**
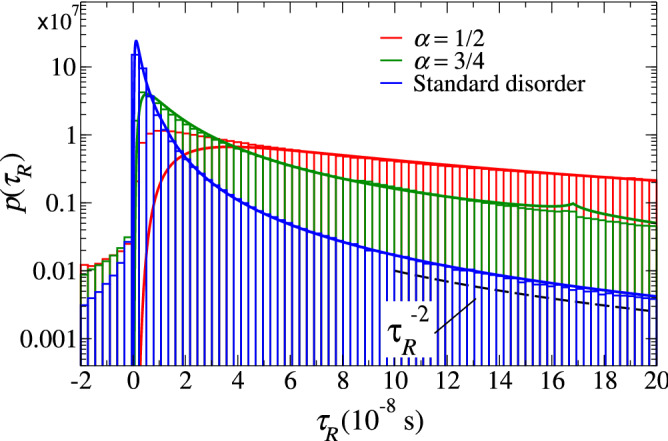
Numerical delay-time distributions with parameters $\alpha =1/2$ (red histogram) and $\alpha =3/4$ (green histogram) and for random waveguides with conventional (Gaussian) disorder (blue histogram). In all cases $\left\langle -\ln T \right\rangle = 10$. The histograms were constructed with $5 \times 10^6$ disorder realizations. The solid curves are the theoretical predictions from Eqs. (3) and (4) for standard and Lévy disorder, respectively. The dashed line, proportional to $1/\tau _R^{2}$, is a guide to the eye.

We now define $s(z,\alpha ,\xi ) \equiv \xi /(2 z^\alpha f(\alpha ))$ with $z=L/(2 n)^{1/\alpha }$ and, for a system of fixed *L*, $\xi =\langle -\ln T \rangle _L$ which is given by^18^: $\langle - \ln T \rangle _L=b L^\alpha f(\alpha )/c$, where $f(\alpha )=(1/2)\int _0^\infty z^{-\alpha } q_{\alpha ,1}(z)dz$. Therefore, using Eqs. (2) and (3), we write the distribution of $\tau _R$ for Lévy disordered samples as

4 $$\begin{aligned} p_{\alpha }(\tau _R)= \int _0^\infty p_{s(z, \alpha ,\xi )}(\tau _R) q_{\alpha ,1}(z) dz , \end{aligned}$$

where $p_{s(z, \alpha ,\xi )}(\tau _R)$ is given in Eq. (3) with *s* replaced by $s(z,\alpha ,\xi )$. The experimental distributions in Fig. 2 have been compared with Eqs. (3, 4) using $\tau _0$ as a fitting parameter.

Numerical simulations are now performed for further support of Eq. (4) and to reveal universal properties. Thus, the number of disorder realizations is greatly increased and absorption, which reduce the effects of long trajectories, is absent. Details of the numerical simulations are provided in the SM.

Figure  3 compares numerical and theoretical delay time distributions from Eqs. (3) and (4) for standard and Lévy disordered waveguides with $\alpha =1/2$ and 3/4 and $\xi =10$. For conventional disorder, our simulations show a physically meaningful result: the absorption time $\tau _0$ can be identified with the average $\langle \tau _R \rangle$. After this identification, since $\langle \tau _R \rangle$ can be extracted from the numerical simulations, there is no free fitting parameters in Eq. (3). Similarly, for Lévy disorder $\tau _0$ has been found to fit the numerical distributions with $\tau _0=2\langle \tau _R \rangle /\alpha$ (for the standard disorder, $\alpha =2$). This result is appealing, however, its formal demonstration remains as an open problem.

Although the distribution profiles in Fig. 3 are different and show the impact of Lévy walks, they share some properties that are not evident because of the different time scale of each case.

**Figure 4:**
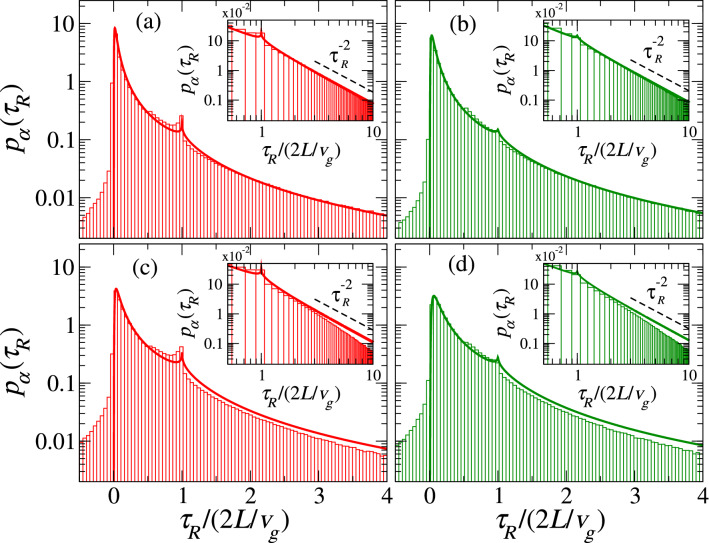
Numerical distributions $p_\alpha (\tau _R)$ (histograms) for Lévy disordered systems characterized by **(a,c)** $\alpha =1/2$ and **(b,d)** $\alpha =3/4$ with **(a,b)** $\xi =10$ and **(c,d)** $\xi =4$. Each histogram was obtained from $5\times 10^6$ disorder realizations. The solid curves are the theoretical predictions from Eq. (4). Insets show $p_\alpha (\tau _R)$ in a logarithmic scale. The dashed lines, following the power law $1/\tau _R^{2}$, are a guide to the eye (see also SM).

In order to compare $p_{\alpha }(\tau _R)$ for different system parameters, it is convenient to express $\tau _R$ in units of $2L/v_g$, as shown in Fig. 4 for $\alpha =1/2$ and 3/4, left and right panels, respectively, and $\xi =10$ and 4, upper and lower panels, respectively. A notorious difference with respect to the distribution for standard disordered systems (Fig. 3, blue line) is that a peak appears at $\tau _R/(2L/v_g)=1$, which is precisely the time that a wave would spend on traveling back and forth between the boundaries of the waveguide in the absence of disorder.

For shorter systems (Fig. 4c,d), we notice a deviation, mainly at the distribution tails, of the theoretical predictions (solid lines) with respect to the numerical results. Also, the numerical simulations start to deviate from the $1/\tau _R^2$ decay (see insets in Fig. 4c,d), which is expected since the transmission is higher as the waveguide becomes shorter.

We now show that some properties of $\tau _R$ go beyond canonical disorder models. We have already mentioned the inverse square decay of the delay time distributions for $\tau _R \gg 1$ obtained in 1D semi-infinite Anderson localized systems; indeed, such power-law behavior has been explained by resonance models in the localized regime^11,44,49^. For Lévy disordered structures, the insets of Fig. 4a,b compare the tail of the distributions for $\alpha =1/2$ and 3/4, respectively, with $1/\tau _R^{2}$ decay (dashed lines); see also the SM for further details and numerical fits of the tails. Actually, from Eqs. (3) and (4), we also find that $p_\alpha (\tau _R) \sim 1/\tau _R^{2}$ for $\tau _R\gg 1$.

**Figure 5:**
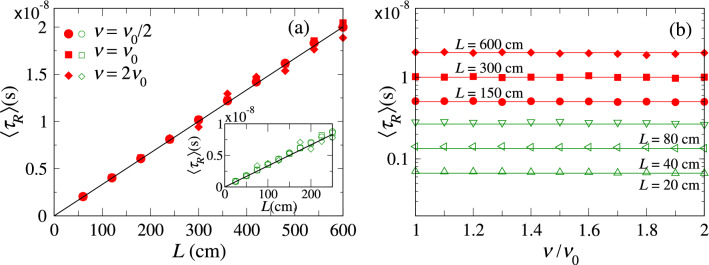
**(a)** Numerical average delay-time $\langle \tau _R \rangle$ for Lévy disorder as a function of the length *L* for different linear frequencies $\nu$ ($\nu _0=7.5$ GHz) with $\alpha =1/2$ (main frame) and $\alpha =3/4$ (inset). **(b)** $\langle \tau _R \rangle$ as a function of $\nu$ for several lengths *L* for $\alpha =1/2$ (red symbols) and $\alpha =3/4$ (green symbols). The averages are obtained from $10^5$ disorder realizations. The horizontal solid lines correspond to $\left\langle \tau _R \right\rangle = L/v_g$ in each case.

Additionally, the average $\langle \tau _R \rangle$ is a linear function of the system length: $\langle \tau _R \rangle = L/v_g$, as shown in Fig. 5a for $\alpha =1/2$ and 3/4 (inset), which is also observed in standard disordered systems^11,43^. Furthermore, an interesting invariance property of the mean length of random walk trajectories with respect to the details of the disorder^16^ has been recently investigated in optical experiments^17^. The invariance of the mean path length is equivalent to the independence of the average delay-time to the energy. We have already shown experimental evidence of this invariance in Fig. 2c,d. Figure 5b provides additional numerical evidence by showing $\langle \tau _R \rangle$ for disordered systems of different lengths with $\alpha =1/2$ and 3/4. We observe that $\langle \tau _R \rangle$ is constant with the linear frequency $\nu$. Thus, these results give further evidence that the invariance of the mean path length goes beyond Brownian random walk models. This invariance can be explained by a direct relation between the delay time and the density of states^2^, as was studied in^15^ from ballistic to localized regimes.

## Discussion

We have studied experimentally and theoretically the impact of Lévy walks on a fundamental dynamical quantity: the delay time. Although the delay-time distributions for Lévy and canonical disordered structures are different, remarkably, some properties are invariant, e.g., the inverse square power-law decay of the delay-time distribution and the insensitivity of the average delay time to energy. The latter is equivalent to the invariance of the mean path length observed in experiments of light propagation. Additionally, the linear dependence of the average delay time with the length in ordinary disordered media is not affected by the presence of Lévy walks. Altogether, our results reveal a universal character of wave propagation that goes beyond standard Brownian models^15–17^.

We point out that in Lévy disordered systems with $\alpha <1$, the mean free path is meaningless since it diverges, in contrast to canonical disordered systems in which the mean free path settles the wave statistics. In the presence of Lévy type of disorder, two quantities determine the transport statistics: $\alpha$ and the logarithmic transmission average.

Our model shows a good agreement with experimental and numerical results, however, only structures with a single transport channel or 1D systems have been considered. Although nowadays 1D wave transport is of relevance, it would be desirable to extend our study to higher dimensions. Nevertheless, the ideas and results presented here are so general that they can be applied from classical to quantum waves in disordered media.

## Methods

To determinate experimentally the delay times $\tau _R$, we employ the experimental setup schematically described in Fig. 1. We use a 2 meters long aluminum waveguide with a rectangular cross-section (22.8 mm width and 10.6 mm height) in which we insert a random distribution of dielectric scatterers. Each scatterer consists of a 2.5 mm thick dielectric slab. The slabs are made of FR4, a composite material with low losses, its dielectric permittivity is $\epsilon =4.4+i0.088$.

With those dimensions of the waveguide, the fundamental mode TE10 propagates along the waveguide in the frequency range from 7.5 to 15 GHz. Within this frequency window, the waveguide can be effectively considered as a 1D random waveguide. A two-port vector network analyzer (VNA) ZVA 24 from Rohde & Schwarz (2 in Fig. 1) with a resolution of 1 Hz is employed to generate and receive microwave signals in the region of interest. The ports of the VNA are used alternatively as sources and receivers of the microwave signals. Each port is connected to the aluminum waveguide by means of standard X-band microwave transitions placed at both sides of the waveguide. This allows the measurements of the four matrix elements of the complex *S*-matrix, $S(\nu )$, where $\nu$ is the linear frequency. In particular, the phase of the reflection amplitude $\theta _R(\nu )$ is obtained as given in Eq. (1).

From the set of measured phases $\theta _R(\nu _i)$, we compute the delay times $\tau _R(\nu ) = d\theta _R(\nu )/d\omega$ as

$$\begin{aligned} \tau _R(\nu _i) \approx \frac{1}{2\pi }\frac{\theta _R(\nu _{i+1})-\theta _R(\nu _i)}{\Delta \nu }, \end{aligned}$$

where $$\Delta \nu =(\nu _{i+1}-\nu _{i})=0.01$$GHz.

We have built waveguides with both Lévy and canonical types of disorder. For the Lévy waveguides, the dielectric slabs have been placed accordingly to a $\alpha$- stable distribution with parameters $\alpha =1/2$ and 3/4, i.e., $\rho (d)\sim 1/d^{1+\alpha }$ for $d\gg 1$. Let us comment that in general there are no closed-form expressions in terms of elementary functions for the Lévy $\alpha$-stable distributions. An explicit closed expression for $q_{\alpha ,c}(x)$ can be conveniently given using the Fourier transform^50^: ${\bar{q}}_{\alpha ,c}(k) =\exp {\left[ -|k|^\alpha \left( B H(k)+{\overline{B}} H(-k) \right) \right] }$, where *H* is the Heaviside step function and ${\overline{B}}$ is the complex conjugate of $B=-c \Gamma (-\alpha )\exp {\left( i\pi \alpha /2 \right) }$, $\Gamma$ being the Gamma function. For any value of $\alpha$ with $0< \alpha <2$, the Lévy $\alpha$-stable distribution is generated numerically^33^.

For the canonical disorder, the separation between slabs follows a standard normal distribution (zero mean and unit variance).

An ensemble of 135 random waveguides of different disorder realizations have been built for each case: $\alpha =1/2$, 3/4, and standard disorder. We measured the reflection amplitude in a frequency window centered at $\nu =9.9$ GHz for Lévy disordered waveguides with $\alpha =1/2$. For $\alpha =3/4$ and for conventional disorder, the reflection amplitude was measured in a frequency window centered at $\nu =11.2$ GHz. Thus, the experimental histograms of Fig. 2a,b have been obtained from 4590 and 1890 delay times, respectively.

## Supplementary information

Supplementary information.
