# Supplementary material for: Delay time of waves performing Lévy walks in 1D random media
Source: Sci Rep. 2020 Nov 30;10:20816. doi: 10.1038/s41598-020-77861-x (PMC7705700; doi:10.1038/s41598-020-77861-x)
Supplement: Supplementary file 1 — Supplementary information. [file 41598_2020_77861_MOESM1_ESM.pdf]

Supplemental Material for *Delay time of waves performing Lévy walks in 1D random media*

L. A. Razo-López[1,+], A. A. Fernández-Marín[1], J. A. Méndez-Bermúdez[1], J. Sánchez-Dehesa[2], and V. A. Gopar[3,*]

[1]Instituto de Física, Benemérita Universidad Autónoma de Puebla, Apartado Postal J-48, Puebla 72570, Mexico
[2]Departamento de Ingeniería Electrónica, Universitat Politècnica de València, Camino de vera s. n. (Edificio 7F), ES-46022, Valencia, Spain
[3]Departamento de Física Teórica, Facultad de Ciencias, and BIFI, Universidad de Zaragoza, Pedro Cerbuna 12, ES-50009 Zaragoza, Spain
[+]Present address: Universitè Côte d'Azur, CNRS, Institut de Physique de Nice, Parc Valrose. 06100 Nice, France
[*]Email: gopar@unizar.es

ABSTRACT

An experimental verification of the distribution of the reflection obtained from the Laguerre ensemble is shown. Also, we provide details of the numerical simulations whose results are shown in the main text as well as numerical verifications of the theoretical distribution of the delay time for canonical disorder, Eq. (3) in the main text.

1 Experimental verification of the Laguerre ensemble: distribution of the reflection

Since the starting point of our model for the delay-time distribution is the Laguerre ensemble, which is formally obtained in the limit of large disordered samples, we have verified that it still works well for our 2m long waveguide. The Laguerre ensemble is given in terms of the variable λ as $p(\lambda) \propto \exp(-\gamma\lambda)$, thus using that $\lambda = R/(1-R)$, we find that the normalized distribution of R in the presence of absorption is given by

$$p(R) = \frac{\gamma}{(1-R)^2} \exp\left[-\gamma R/(1-R)\right], \tag{S.1}$$

where γ is the absorption strength. In Fig. S1, we show the experimental distribution (histogram) of the reflection for conventional disordered waveguides and $p(R)$ (solid line) as given by Eq. (S.1), using γ as a fitting parameter. A good agreement is seen. With this verification, we proceeded with our model for the time delay distribution for Lévy waveguides.

2 Numerical verification of Eq. (3)

It is important to verify the distribution $p_s(\tau_R)$ (Eq. (3) in the main text) in ordinary disorder systems since it is used to calculate the distribution of the delay time of Lévy disordered systems. The distribution $p_s(\tau_R)$ is given by (Eq. (3) in the main text):

$$p_s(\tau_R) = \frac{a}{s[2-\exp(-a/s)]} \frac{1}{\tau_R^2} \exp(-a|1/\tau_R - 1|/s),$$

where $s \equiv \langle -\ln T \rangle = L/\ell$ with L and ℓ being the length of the system and the mean free path, respectively, and $a = 2L/(v_g \tau_0)$.

The numerical simulations were performed considering a 1D photonic model, so we compute its transport properties by the use of the transfer matrix method, see Ref.[2] of this SM. As in our experiments, we consider a structure of alternating layers of different index of refraction $n_a = 1$ (air) and $n_b = \sqrt{4.4}$ (FR4 plastic). The separation l_{ja} between dielectric slabs is randomly extracted from a Lévy α-stable distribution with parameter α for Lévy disordered structures and from a Gaussian distribution (mean zero and variance unity) for ordinary disordered samples. The slabs are placed alternating right and left segments of the sample until filling the structure of fixed length. The width of the dielectric slabs is constant, $l_b = 2.5$mm.

The transfer matrix of a single sample of this structure can be written as

$$\mathbf{M} = \mathbf{M}_{Na}\mathbf{M}_b\mathbf{M}_{(N-1)a} \cdots \mathbf{M}_{2a}\mathbf{M}_b\mathbf{M}_{1a}\mathbf{M}_b\mathbf{M}_{0a}, \tag{S.2}$$

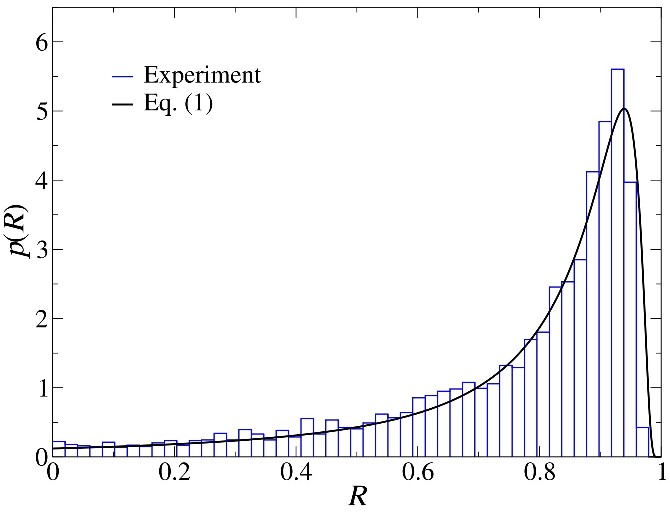

Figure S1. Comparison of the experimental distribution of the reflection $p(R)$ (histogram) for 2m long waveguide with canonical disorder and the theoretical result in Eq. (S.1) with $\gamma = 0.12$.

where

$$\mathbf{M}_{ja} = \begin{pmatrix} \mathrm{e}^{\mathrm{i}k_{za}l_{ja}} & 0 \\ 0 & \mathrm{e}^{-\mathrm{i}k_{za}l_{ja}} \end{pmatrix} \tag{S.3}$$

and the components of the matrices $\mathbf{M}_b$ ($\mathbf{M}_b^{i,j}$) are given by

$$\begin{aligned} M_b^{11} &= \cos k_{zb}l_b + \frac{\mathrm{i}}{2}\left(\frac{k_{zb}}{k_{za}} + \frac{k_{za}}{k_{zb}}\right)\sin k_{zb}l_b, \\ M_b^{12} &= \frac{\mathrm{i}}{2}\left(\frac{k_{zb}}{k_{za}} - \frac{k_{za}}{k_{zb}}\right)\sin k_{zb}l_b, \\ M_b^{21} &= -\frac{\mathrm{i}}{2}\left(\frac{k_{zb}}{k_{za}} - \frac{k_{za}}{k_{zb}}\right)\sin k_{zb}l_b, \\ M_b^{22} &= \cos k_{zb}l_b - \frac{\mathrm{i}}{2}\left(\frac{k_{zb}}{k_{za}} + \frac{k_{za}}{k_{zb}}\right)\sin k_{zb}l_b. \end{aligned} \tag{S.4}$$

Here, k_{zi} is the component of the wave vector along the z axis in the layer i. It is defined as $k_{zi} = (\omega/\mathrm{c})n_i \cos\phi_i$ where ϕ_i is angle of incidence in the medium i. From the transfer matrix $\mathbf{M}$ in Eq. (S.2), we obtain the reflection amplitude:

$$\sqrt{R}\mathrm{e}^{\mathrm{i}\theta_R} = \frac{M^{12}}{M^{22}}. \tag{S.5}$$

Finally, the delay time is given by

$$\tau_R = \frac{\mathrm{d}\theta_R}{\mathrm{d}\omega}. \tag{S.6}$$

In Figs. S2(a) and S2(b) for $s = 10$ and 4, respectively, we compare $p_s(\tau_R)$ given in Eq. (3) and the distribution of the time delay from the numerical simulations. From Fig. S2, a good agreement between the numerical simulations (histograms) and theoretical expression for $p_s(\tau_R)$ (solid curves) is observed. The insets in Fig. S2 show $p(\tau_R)$ in a logarithmic scale together with the power-law tail $1/\tau_R^2$ (dashed lines), expected for semi-infinite systems. Fits of the tail of $p(\tau_R)$, however, show that the power of the tail for standard disorder is -2.24 ± 0.04, while for Lévy disordered structures (as those in Fig. 4 in the main text) is -2.30 ± 0.04 and-2.31 ± 0.04 for $\alpha = 1/2$ and 3/4, respectively. In all these cases, $\langle -\ln T \rangle = 10$. While the values of the power differ approximately 10% from the theoretical value -2, these differences may be due to the finite size of the systems (recall that the power -2 is obtained in the limit of large L). We remark, however, that the power of the tails obtained from the fits are approximately the same for all types of disorder, indicating the insensitivity of the power-law decay to the type of disorder.

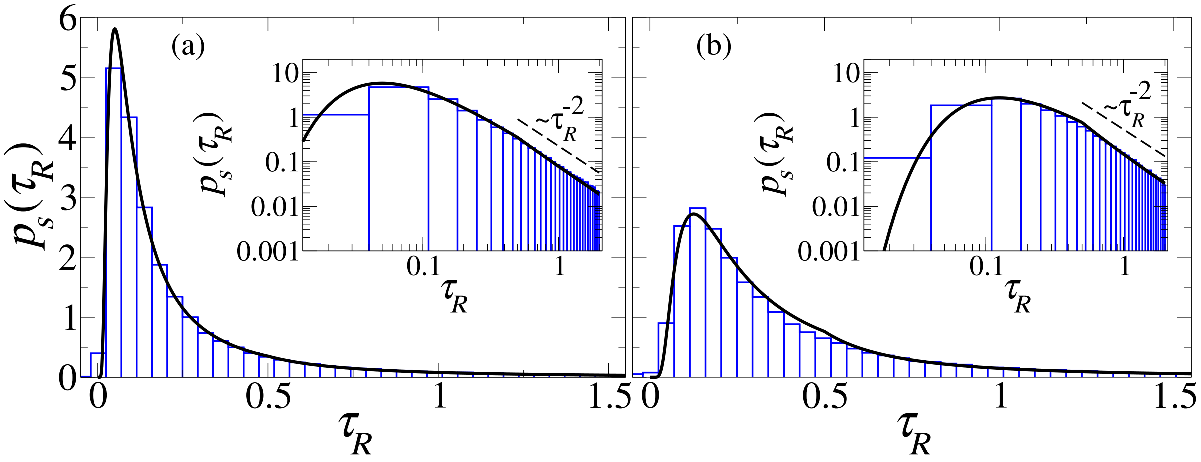

Figure S2. Numerical delay time distributions $p_s(\tau_R)$ (histograms) for random waveguides with standard (Gaussian) disorder characterized by (a) $s = 10$ and (b) $s = 4$. The histograms were constructed with 10^5 disorder realizations. The solid curves are the theoretical predictions from Eq. (3), in the main text. Insets show $p_s(\tau_R)$ in a logarithmic scale. The dashed lines are guides to the eye and follow the law $1/\tau_R^2$.

3 Parameter values of the theoretical distributions

The group velocity v_g in $p_s(\tau_R)$ (in the definition of the constant a) is obtained from $v_g = c/n_{eff}$ where we have considered as effective index of refraction[3] $n_{eff} = (f_a n_a + f_b n_b)$, f_i being the filling factor for the layers with refraction index n_i ($i = a$: air, $i = b$: dielectric). For conventional disorder, we have that $f_a = f_b = 1/2$. For the time of absorption τ_0, we found a good agreement precisely by fixing $\tau_0 = \langle\tau_R\rangle$. Therefore, since $\langle\tau_R\rangle = L/v_g$, $a = 2$, in Figs. S2(a) and S2(b), in this SM.

Interestingly, we have found numerically that the distributions of τ_R are well described by $\tau_0 = 2\langle\tau_R\rangle/\alpha$. For Lévy disorder, we can approximate $f_a \approx 1$. Therefore, $a = \alpha$, in the expression for $p_s(\tau_R)$ in Eq. (3) in the main text; thus, Eq. (4) is plotted in Figs. 3 and 4 (solid lines) in the main text.

So far absorption has not been considered in our numerical simulation, however, experimental results are affected by the presence of absorption. For instance, we have performed numerical simulations with absorption that shown that the average $\langle\tau_R\rangle$ is no longer a linear function of the system length. We have thus introduced a factor in τ_0 to consider the absorption of the experimental waveguides. In this manner, in the theoretical distributions in Fig. 2 (main text), we have used $\tau_0 = 16.3\langle\tau_R\rangle$, $12.6\langle\tau_R\rangle$, and $22.9\langle\tau_R\rangle$ for $\alpha = 1/2,\ 3/4$, and canonical disorder, respectively.

References

1. Uchaikin, V. V. and Zolotarev, V. M., *Chance and Stability. Stable distributions and their applications* (De Gruyter, Uthrech, The Netherlands, 1999).
2. P. Markoš and C. M. Soukoulis, *Wave Propagation. From Electrons to Photonic Crystals and Left-Handed Materials* (Princeton University Press, Princeton, NJ, 2008).
3. J. Carbonell, F. Cervera, J. Sánchez-Dehesa, J. Arriaga, L. Gumen, and A. Krokhin, Appl. Phys. Lett. **97**, 231122, (2010).
